# Digital health RCT interventions for cardiovascular disease risk reduction: a systematic review and meta-analysis

**DOI:** 10.1007/s12553-022-00651-0

**Published:** 2022-03-25

**Authors:** Rohan Neil Devani, Arushan Kirubakaran, Mariam Molokhia

**Affiliations:** Department of Life Sciences and Medicine, King’s College London, Great Maze Pond, London, SE1 1UL UK

**Keywords:** Digital health, eHealth, mHealth, Digital technology, Cardiovascular disease

## Abstract

Heart disease is a leading cause of UK mortality. Evidence suggests digital health interventions (DHIs), such as smartphone applications, may reduce cardiovascular risk, but no recent reviews are available. This review examined the effect of DHIs on cardiovascular disease (CVD) risk scores in patients with increased CVD risk, compared to usual care alone. PubMed, Cochrane Database, Medline, and Google Scholar were searched for eligible trials published after 01/01/2010, involving populations with at least one CVD risk factor. Primary outcome was change in CVD risk score (e.g. QRISK3) between baseline and follow-up. Meta-analysis was undertaken using Revman5/STATA using random-effects modelling. Cochrane RoB-2 tool determined risk-of-bias. 6 randomised controlled trials from 36 retrieved articles (16.7%) met inclusion criteria, involving 1,157 patients treated with DHIs alongside usual care, and 1,127 patients offered usual care only (control group). Meta-analysis using random-effects model in STATA showed an inconclusive effect for DHIs as effective compared to usual care (Mean Difference, MD -0.76, 95% CI -1.72, 0.20), with moderate certainty (GRADEpro). Sensitivity analysis by DHI modality suggested automated email messaging was the most effective DHI (MD -1.09, 95% Cl -2.15, -0.03), with moderate certainty (GRADEpro). However, substantial study heterogeneity was noted in main and sensitivity analyses (I^2^ = 66% and 64% respectively). Quality assessment identified risk-of-bias concerns, particularly for outcome measurement. Findings suggest specific DHIs such as automated email messaging may improve CVD risk outcomes, but were inconclusive for DHIs overall. Further research into specific DHI modalities is required, with longer follow-up.

**Supplementary Information:**

The online version contains supplementary material available at 10.1007/s12553-022-00651-0.

## Introduction and rationale

Cardiovascular disease (CVD) [1] contributes to 27% of mortality in the UK annually [2] and is the most prevalent cause of global mortality [3]. CVD is devastating to those over 75 years in the UK, causing 120,000 deaths a year – three-quarters of total CVD mortality [2] with annual cost of £21.9 billion, [4]. Public Health England recommends novel approaches in treating the national burden of CVD as a matter of urgency. Current therapies include lifestyle measures, anti-hypertensives, and lipid modification therapies, [4] as recommended by the UK National Institute for Health and Care Excellence [1] and outlined as a priority in the NHS Long-Term Plan [5]. Barriers to implementation of these targets include patient factors such as inequalities in healthcare access [6] and lack of education on diet and lifestyle [6], and staff factors: personnel shortages, limited resources and time for health promotion with patients, [6] and high workload [7], which can result in poorer quality of care.

Digital health interventions (DHIs) are technology-based solutions aiming to improve health and treatment of patients efficiently and safely [8]. Examples include smartphone applications and automated email services. Research by Whittaker and Wieland found DHIs could reduce cardiovascular risk factors, such as smoking cessation [9] and weight loss, [10] but it is unclear whether these benefits are sustainable long-term. Marvel indicated DHIs, such as smartwatches, smartphone applications and blood pressure monitors are effective in preventing CVD events, particularly the recurrence of myocardial infarction in high-risk populations [11]. However, this study was only conducted in the USA, a high-income country, and results may not be generalisable globally. Rawstorn found evidence that digital interventions (such as automated email reminders and online educational patient tutorials) could improve modifiable risk factors such as LDL-cholesterol and diastolic blood pressure [12]. However, only 11 studies were reviewed, and participants had low-to-moderate cardiovascular risk, therefore results may not be applicable to high-risk CVD populations.

There is limited research in this area – the most recent evidence on DHIs and CVD risk reduction is Widmer’s 2015 systematic review [13]. Widmer’s meta-analysis found DHIs reduced rehospitalisation rates and improved Framingham risk score (relative risk: 0.61; 95% CI 0.46, 0.80), while facilitating reductions in body mass index (BMI) and weight. The utility of the study was limited by moderate study heterogeneity (*I*^*2*^ = 22%), that could not be accounted for by study design, reducing generalisability of the results. Another systematic review of 7 studies similarly found DHIs (such as smartphone and web applications) were both feasible and acceptable for patients in reducing CVD risk factors via cardiac rehabilitation when undertaken remotely [14]. However, these findings are limited as only qualitative results were reported – and lacked predictive frameworks for modelling patient management or ensuring improved outcomes [15].

Van Halewijn’s review of all-cause and cardiovascular mortality, MI and cerebrovascular events, found studies using two complementary interventions, such as online tutorials and activity logging combined with email feedback were effective in reducing CVD events. The multi-disciplinary approaches from studies included in the review reduced CVD mortality, myocardial infarction and cerebrovascular events [16]. However, this approach requires co-ordinated activity across the treatment team to deliver effective care [17].

Digital health technologies have improved exponentially in power and complexity over the past decade [18]. This review offers an updated evaluation of recently-developed DHIs and their effect on CVD risk score, to guide management of CVD in primary care, in high-risk patients.

## Methodology

This systematic review followed PRISMA (Preferred reporting items for systematic reviews and meta-analyses) guidelines [19], and was accepted by PROSPERO, an international database of systematic review protocols (reference: CRD42021236963, accepted 31/03/2021).

### Search strategy

**Fig. 1:**
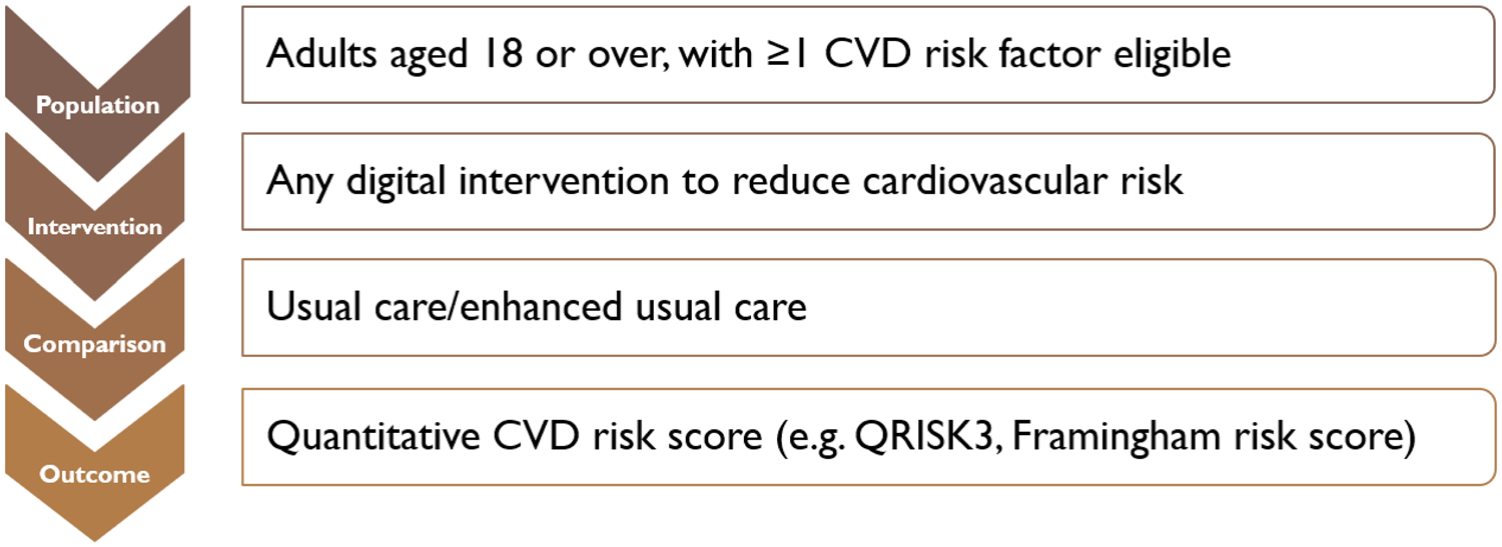
Population, intervention, comparison, outcome statements for this systematic review

Previous high-quality systematic reviews on CVD and DHIs were analysed to determine search terms for databases [13, 14]. A PICO (population, intervention, comparison, outcome) framework was developed to refine search terms (Fig. 1). A search strategy of specific MeSH (medical subject heading) terms was formulated, relating to CVD and DHIs. Further information is given in Supplementary information.

Four electronic databases were searched on 01/03/21: PubMed, Cochrane Database of Systematic Reviews, Medline, and Google Scholar.

### Study selection and eligibility criteria

The study population included individuals 18 years or older, with at least one CVD risk factor as defined by NICE guidelines [1]. This included: history of previous CVD event such as stroke, being overweight, amongst others.

All participants in included studies had at least one cardiovascular risk factor (such as diagnosis of hypertension), or previous CVD event such as stroke. Participants in each study also received guidance to ensure sufficient understanding of the DHI to engage effectively with the intervention.

Interventions included DHIs to reduce CVD risk, such as web applications. The control group was usual care. Outcomes considered were clinically-validated international recognised CVD risk scores, such as QRISK3 [20] or Framingham 10-year risk score [21]. These scores are calculated via an algorithm considering risk factors such as gender, smoking status, and total cholesterol to synthesise a score expressing the likelihood of a cardiovascular event occurring. Studies which did not report clinically-validated CVD risk scores were excluded. For example, studies measuring composite outcome scores of several selected risk factors (smoking cessation, BMI etc.) were not included.

Only English-language randomised controlled trials, with full results published after 1^st^ January 2010 were considered. There was no exclusion by setting (whether population- or care-based).

Two authors (RD, MM) independently reviewed the selection of papers and appropriateness for inclusion.

### Study screening

Search results were imported into the Rayyan application [22] for screening by RD. Duplicates were removed, followed by a round of initial screening of abstracts and titles by RD and AK. Full text of remaining articles were screened, and reasons recorded for excluded studies. A second reviewer (MM) assessed remaining articles and disagreements were resolved by discussion.

The bibliography of included articles were scanned to find further eligible papers. For 3 papers, full text could not be retrieved, and contacting the author was unsuccessful.

### Data extraction

RD independently extracted data from selected papers. First author, year, setting, study design, follow-up duration, DHI method utilised, control group, outcome measure, confounders adjusted for, fully-adjusted outcome score and funding were all summarised in Table 1, according to Cochrane guidance [23].

### Statistical calculations of results

Meta-analysis was conducted to estimate mean difference in CVD risk score (95% confidence interval) using internationally-recognised CVD scoring systems. Random-effects modelling facilitated measurement of the mean of distribution of several effects, allowing for study heterogeneity, rather than a single true effect size (in fixed-effects modelling) [24] using Revman5 software to generate a forest plot [25]. Statistical heterogeneity between the studies was measured by *I*^2^ statistic; *Tau*^***2***^ was reported as a measure of study variance.

Meta-analysis and subsequent subgroup analysis of mean effect change by DHI modality (automated email combined with web application) was undertaken.

## Results

### Studies included for analysis

**Fig. 2:**
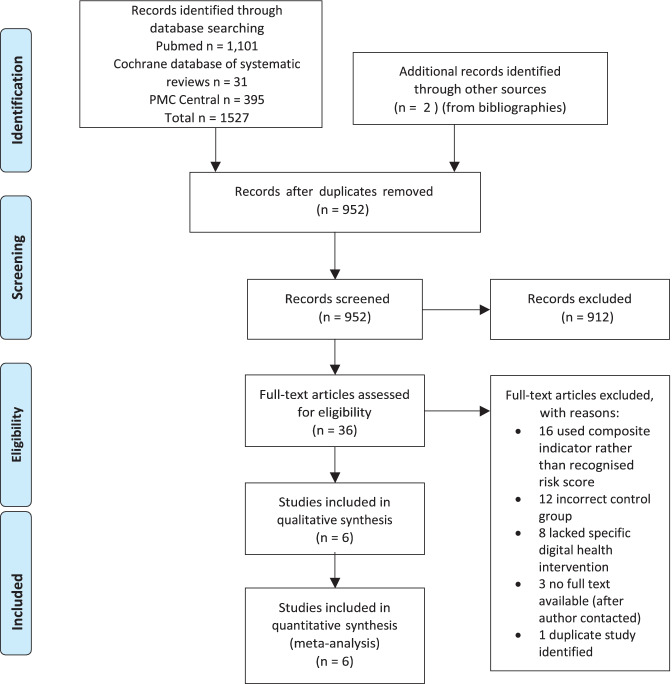
PRISMA flowchart of study selection for this review (Moher et al. [19])

1,527 studies were identified on searching electronic databases for title and abstract screening. 36 studies were selected for full-text review. Finally, six studies (6/36 = 16.7%) from 5 countries were considered eligible for analysis and selected for review. The studies comprised 2,284 individuals total, divided into 1,157 subjects in DHI intervention groups, and 1,127 subjects assigned to usual care (control groups). The full process of study selection is illustrated in Fig. 2.

**Fig. 3:**
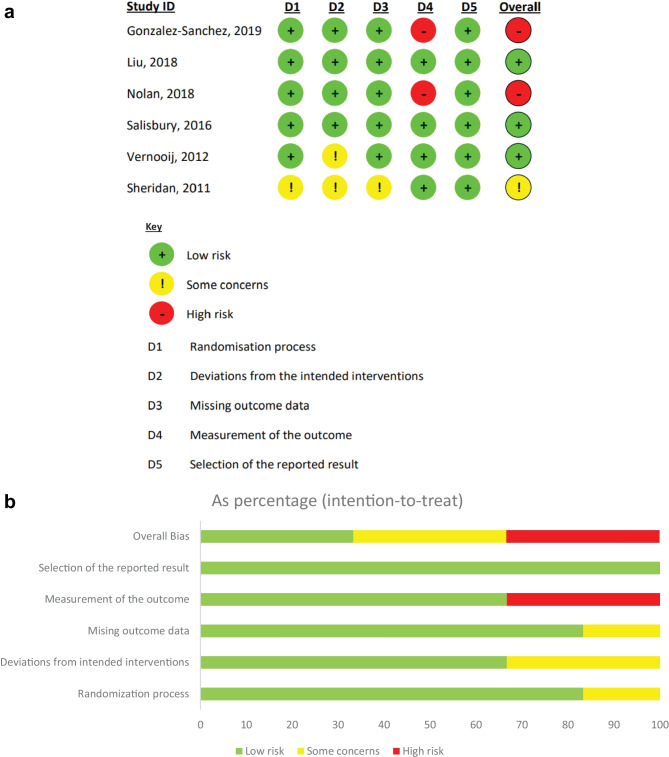
**a** Results of analysis of bias, conducted using the Cochrane Risk-of-bias 2 (RoB-2) tool. **b** Overall results of Cochrane Risk-of-Bias analysis, with bias in each domain expressed for all included studies of this review

Framingham Risk Score (FRS) was the most common risk score—reported in 4 of the included studies; D’Agostino risk score (based on FRS) and QRISK2 (an earlier version of QRISK3 previously used in UK Primary Care) reported by Gonzalez-Sanchez [26] and Salisbury [27] respectively. The mean follow-up duration overall was 9.2 months (range 3–12 months).

The intervention arm of 4 studies utilised dual DHI modalities involving automated email alongside a web application accessible to intervention group participants. Vernooij’s study [28] involved a web application alone, Gonzalez-Sanchez’s [26] study involved a smartphone application alone.

None of the studies reported socio-economic or ethnic demographic data. Studies recruited participants through either primary (3 studies) or secondary care providers (1 study) or through an online website (2 studies).

### Quality assessment of reported studies

Two authors (RD, MM) independently reviewed risk of bias using Cochrane Risk-of-bias 2 (RoB-2) tool to each study included in the review [29]. Included papers were rated ‘high risk’, ‘low risk’ or ‘some concerns’ across five domains (Fig. 3a, b). Assessment revealed overall ‘high risk’ of bias in 2 studies – due to domain 4 (measurement of outcome). A judgement of ‘some concerns’ was synthesised for overall bias.

Domains recorded included: randomisation process, deviations from the intended outcomes, missing outcome data, measurement of the outcome and selection of the reported result. Guidance in determining risk of bias in each domain was provided by the accompanying handbook provided by Cochrane [30]. On comparing discrepancies between results, the handbook was consulted and disagreements resolved, leading to the production of Fig. 3a, b.

### Synthesis of results

**Fig. 4:**
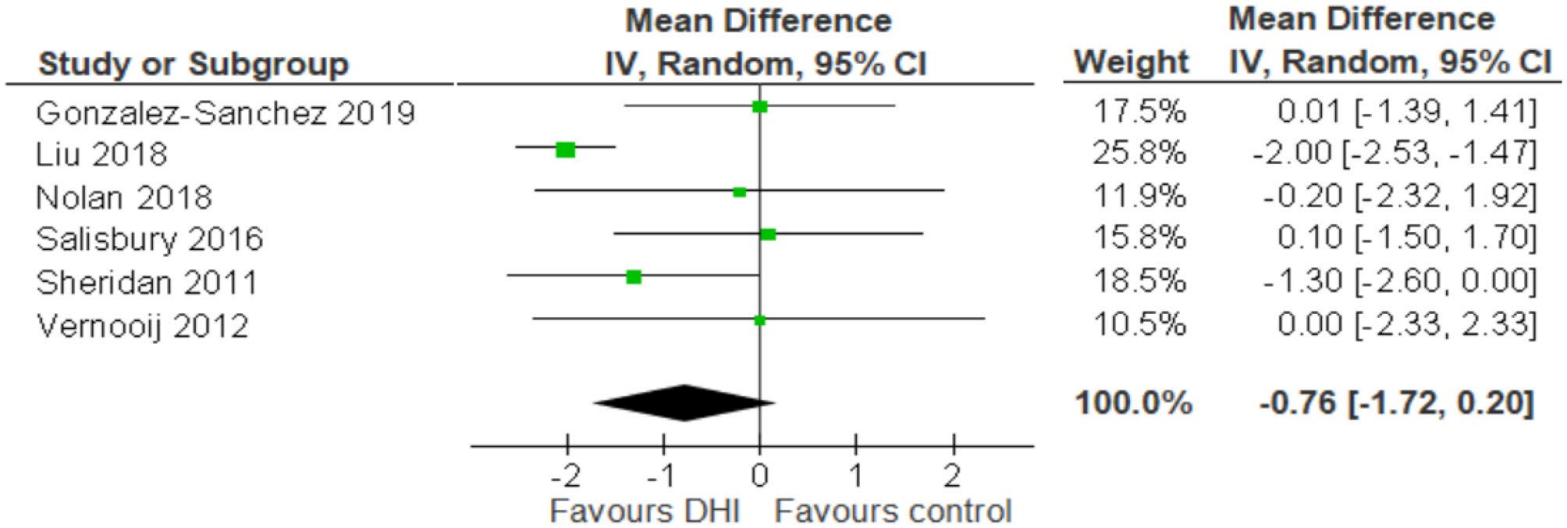
Forest plot showing meta-analysis of the six included studies

Random-effects meta-analysis and forest plot (Fig. 4) showed inconclusive evidence that utilising DHIs alongside normal care can improve CVD risk score (MD -0.76, 95% CI -1.72, 0.20), rated with “moderate” certainty by GRADEpro scoring [31]. However, these results may not be generalisable to other settings, due to substantial heterogeneity between studies (*I*^*2*^ = 66%). Moreover, variance between true effects was relatively high (*Tau*^*2*^ = 0.85) (Fig. 4).

Papers presented adjusted absolute differences (n = 2) or mean differences in CVD risk score (n = 4). 4 papers measured effect size as mean difference between control and intervention group at follow up [26, 27, 32, 33]. Sheridan calculated adjusted absolute difference [34]. Vernooij measured relative change in synthesised risk score by subtracting change in intervention group score from baseline, subtracted by change in usual care group from baseline, with this figure divided by mean risk score at baseline [28].

### Subgroup analysis: automated email messaging with web applications

**Fig. 5:**
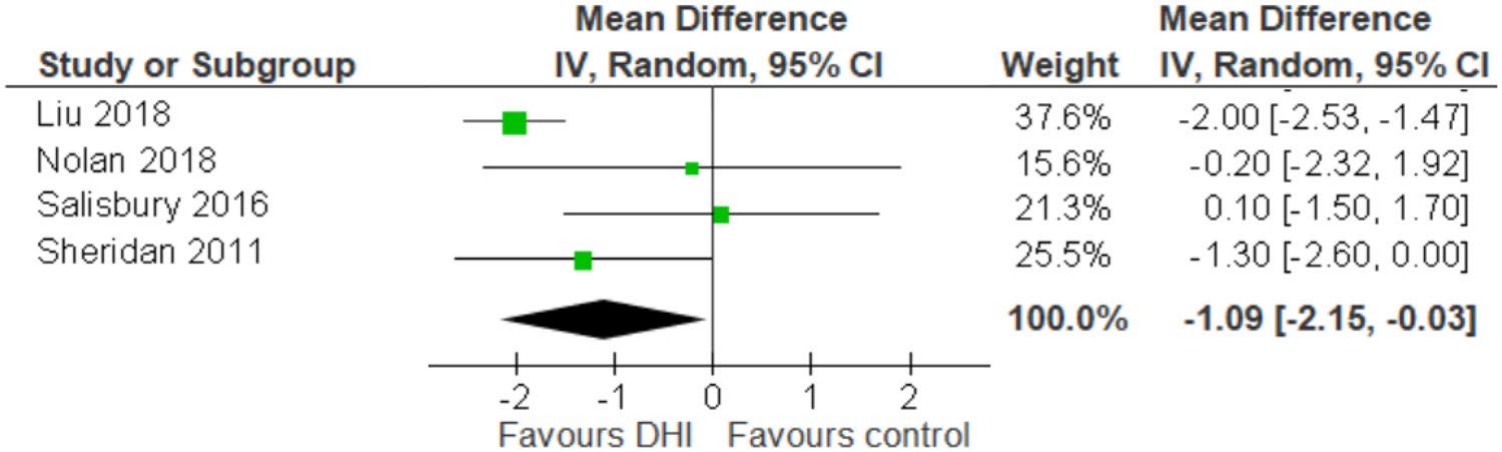
Forest plot showing subgroup analysis of the four studies utilising automated email messaging and web application DHIs together

4 of the 6 studies provided data on automated email messaging combined with a web application. Pooled subgroup analysis on these studies (Fig. 5) suggested this DHI methodology may reduce CVD risk (MD: -1.09, 95% Cl -2.15, -0.03). However, heterogeneity of these studies remained high: *I*^*2*^ = 64%, similar to the main analysis (Fig. 4).

### Results in the context of methodological quality of the included studies

Methodological quality was assessed by RoB-2. Table 1 shows where participants were recruited from. Eligibility criteria of included studies were checked extensively to ensure participants were suitable for this review’s criteria (as detailed in Sect. 2.2). The method of randomisation of participants to either the control or intervention group was named in 5 of the 6 studies (Sheridan’s excluded [34]). Blinding of group allocation occasionally could not be maintained for participants due to the nature of the interventions, but most research groups (n = 5) were single-blinded at least.

**Table 1:** Data extraction table of included studies

First author, Year	Country	Study Design	Sample size (intervention group)	Setting	Follow-up duration	DHI method utilised (intervention group)	Control group	Outcaome measure	Fully adjusted mean difference (95% CI)	Confounders adjusted for	Funding
Gonzalez-Sanchez et al. [26]	Spain	RCT	833 (n = 415)	General population recruited from Primary Care	12 Months	Smartphoneapplication + in-person counselling	In-person counselling only	D’Agostino	0.01 (-1.39 to 1.41)	Outcome adjusted for anti-hypertensive drugs, lipid-lowering drugs	Spanish Ministry of Science and Innovation and Carlos III Health Institute/European regional Development Fund; and the Regional Health Management of Castilla and León
Liu et al. [33]	Canada	RCT	86 (n = 43)	General population, primary prevention, recruited online	4 months	Automated messaging (Email), Web-based application	Usual care	FRS	-2.00 (-2.53 to -1.47)	Outcome controlled for baseline CVD risks	Heart and Stroke Foundation of Canada, Focus on Stroke Award and Canadian Institute of Health research
Nolan et al. [32]	Canada	RCT	240 (n = 133)	Multi-centre population-based study, recruited online	12 months	Automated messaging (Email), Web-based application	Usual care + self care education	FRS	-0.20 (-2.32 to 1.92)	Outcome adjusted for baseline CVD risk, gender and medication	Canadian Institutes of Health Research
Salisbury et al. [27]	England	Pragmatic RCT	641 (n = 325)	General population recruited from Primary Care	12 Months	Automated messaging (Email), Web-based application	Usual care	QRISK2	0.10 (-1.50 to 1.70)	Outcome adjusted for baseline CVD risk smoking history and General practice	National Institute for Health research (NIHR)
Vernooij et al. [28]	Netherlands	RCT	330 (n = 164)	Secondary and Tertiary care	12 months	Web-based application	Usual care	FRS	-1.30 (-2.60 to 0.00)	Outcome controlled for baseline CVD risk	ZonMw, the Netherlands Organization for Health Research and Development
Sheridan et al. [34]	USA	RCT	154 (n = 77)	General population recruited from Primary Care	3 months	Automated messaging (Email), Web-based application	Usual care	FRS	0.00 (-2.33 to 2.33)	Outcome adjusted for baseline CVD risk, education level	American Heart Association, the National Heart Lung and Blood Institute, and the National Cancer Institute

*FRS* 10-year Framingham risk score, *DHI* Digital health intervention, *RCT* randomised controlled trial

DHI intervention fidelity of delivery and uptake was reported in multiple studies. Salisbury’s web application and email reminder system [27] also logged interaction levels per participant, to gauge engagement with the intervention. Gonzalez-Sanchez’s mobile phone application [26] was accompanied by a phone call 15 days after trial commencement to ensure the application was being used correctly. Clinical assessments were offered at studies where repeated follow-up measurements were taken. N = 3 trials did not mention monitoring participants’ engagement with the interventions, limiting the significance of the trial’s results.

Table 2 provides further details on the specific DHIs utilised and how they were offered to participants.

**Table 2:** Details on methodology of interventions in included studies

First author, YHr	DHI method utlUsed (lni-ntlon group)	Control group	Trial and DHI details
Gonzalez-Sanchez et al. [26]	Smartphone application + in person counselling	In-person counselling	A smartphone application was developed by software designers and dieticians. The application offered functionality to record food intake, and utilised the smartphone's accelerometer to calculate physical activity such as step count. Trial parrticipants were also instructed to use the application regularly and record data to ensure engagement
Liu et al. [33]	Automated messaging (Email), Web-based application	Usual care	Participants underwent pre-trial clinical assessment, and communication with participants' physicians was sought. From this data, a user-driven web-application was created offering governmental health guidance, and information on exercise and diet plans. Emails containing dietary and exercise plans were also sent to participants
Nolan et al. [32]	Automated messaging (Email), Web-based application	Usual care + self care education	Communication with participants' physicians and assessment of participants' English language skills was sought. A web application for participants offered links to health resources. Self-monitoring tools were also provided, such as interactive forms to track progress. The transtheoretical model of cognitive behavioural change was used to help participants create targets for themselves
Salisbury et al. [27]	Automated messaging (Email), Web-based application	Usual care	Health advisors supported participants via interactive software with interactive, computerised scripts to help them set individual health goals. Participants could also access the 'Healthlines' web portal to learn about CVD. Blood pressure and other metrics could be uploaded to the Healthlines portal, which calculated an average of the readings and offered automated advice to participants
Vernooij et al. [28]	Web-based application	Usual care	A website application was personalised for individual patients, based on their cardiovascular risk. Patients could submit blood pressure, weight, smoking and cholesterol levels. Messages could also be sent and received between themselves and nurse practitioners. The application also offered links to further information on vascular diseases and health information
Sheridan et al. [34]	Automated messaging (Email), Web-based application	Usual care	A decision aid was provided to participants encouraging them to reduce their CVD risk, and counselled them in communicating with their physician regarding CVD. The tailoredtext- messaging system aimed to develop participant skills in overcoming barriers to exercise and healthy eating. The messaging system included a collection of 76 unique messages. This could be adjusted with over a million combinations tailored to individual participants dependant on their answers to an initial set of survey questions

## Discussion

### Discussion of results

This review found six studies evaluating the effect of DHIs on CVD risk scores, three papers with results favouring DHIs, and three papers showing no evidence that DHIs improved CVD risk score compared to a control group of usual care. Ultimately, the results (Mean Difference -0.76, 95% CI -1.72, 0.20), with moderate GRADEpro certainty, showed inconclusive evidence in favour of DHIs reducing risk score, despite higher weighting for the two larger studies (Liu, Sheridan). Consistency in the results of protective studies were associated with shared DHI modality.

Subgroup analysis of trials involving automated messaging (via email) with a complementary web application suggested evidence of a protective effect (MD: -1.09, 95% Cl -2.15, -0.03) [27, 32–34]. The findings of Nolan [32], Liu [33] and Sheridan [34] found improvements in CVD risk score after varying follow-up durations (range 3–12 months). Two of these studies had follow-up longer than average (9.16 months), although shorter studies may have not allowed sufficient opportunity for an intervention effect (Fig. 4).

Of the studies which showed a reduction in CVD risk [32–34] participants were recruited either from their primary care providers or online websites, including equal proportion of genders – apart from Sheridan’s [34] study where one-quarter of subjects were female. Liu’s findings showed the greatest reduction in CVD risk, and low risk of bias. The confidence interval (MD: -2.00, 95% CI -2.53, -1.47) suggested high study precision. This trial split participants into three groups – offering an email newsletter combined with personalised exercise and diet prescription, email newsletter alone, or a control group allocated to usual care. Both groups receiving email had greater mean difference from baseline risk score than control group at 12 months follow-up.

Liu’s 2018 paper [33] had the greatest effect size, despite a small sample size (86 participants, n = 43 in intervention arm). The weighting of this study was 25.8%, (using Revman 5 software), which weights studies according to the reciprocal of their variance [35]. This reflected the high level of precision in the study. Substantial heterogeneity (*I*^*2*^ = 66% and 64%) between main and sensitivity analyses suggests results are limited in generalisability to other healthcare systems, potentially due to the confounders adjusted for in each individual study, and differences in methodology. This is despite applying a random-effects statistical model to account for a high degree of variance. However, this heterogeneity finding should be considered in the context of evidence suggesting larger sample sizes may influence and inflate the I^2^ result [36].

The analyses suggested effect sizes differed across the studies, with substantial study heterogeneity (I^2^ 66%). Digital health interventions to improve cardiovascular health are relatively new technologies, and hold promise for the future. As such, new applications and programmes to improve or rehabilitate cardiovascular health are regularly being developed, particularly following the advent of remote medicine, further accelerated by the COVID-19 pandemic. However, there remain few trials assessing the effectiveness of DHIs on CVD patients. The review included studies with comparable patient populations, and each study involved patients with a history of at least one CVD event (such as stroke) or CVD risk factor such as hypertension. Moreover, the trials were all conducted in developed countries with comparable standards yet diverse healthcare systems, all ranking inside the top 22 countries for outcome of cardiovascular disease care, as per the OECD ranking system [37].

### Results of this review in the context of similar studies

The King’s Fund has identified emerging major trends in new health technologies being developed and trialled at a rapid rate [38], meaning review of new, unanalysed DHIs is warranted. Many studies consider the effect of DHIs on specific CVD risk factors, but studies on CVD risk scores are lacking, apart from Widmer who found DHIs reduce CVD risk score and positively impact risk factors such as BMI [13]. Although a research protocol for an upcoming systematic review considering a range of CVD risk scores and risk factors has been published [39] although results are unpublished.

Of those examining risk factor reduction alone, a systematic review found behavioural counselling DHIs improved blood pressure control [40]. However, the study population was skewed towards male participants (66%). Representative solutions, where men and women are enrolled in trials at similar rates, are needed to ensure an accurate evidence base when devising CVD treatment plans [41]. Aronow’s systematic literature review based on 58 RCTs found web-based applications decrease all-cause mortality and heart-failure related hospitalisations [42]. Mean age was not recorded; importantly RCTs which enrolled older patients found significant improvements in all-cause mortality. Included studies were focused on community-dwelling patients, and patients monitored as part of transitional care, beginning in the hospital setting. These findings are indicative of a wider trend suggesting novel technologies reduce CVD-related rehospitalisation, but this research is limited as the most effective DHI modality is yet to be identified.

An RCT focusing on a target population of pre-diabetic patients with an intended goal of weight loss (ineligible for inclusion in this review), suggested automated email is a successful, scalable approach to reach millions of patients and reduce CVD risk factors such as weight loss [43]. Unfortunately, there is limited systematic evidence synthesis on the effect of automated email in healthcare. However, Dalal’s findings predicted automated emails could, in future, notify patients of healthcare results [44]. Although neither of these studies focused on CVD prevention specifically, automated messaging DHIs have potential healthcare applications. Currently patient contacting is widely undertaken through automated and bespoke text messaging systems in UK primary care [45], but this solution depends on access to mobile phones.

Van Halewijn’s 2017 review analysed cardiovascular mortality rates in studies where DHIs including online tutorials were offered. Data on prevalence of secondary outcomes such as MI and cerebrovascular events were also extracted, which are relevant consequences of CVD. Van Halewijn found DHIs as part of comprehensive cardiac rehabilitation reduces all-cause mortality in patients with higher risk of CVD [16]. However, loss to follow-up in this study was high, meaning that the results could be biased as advantages of cardioprotective DHIs could be overstated.

### Strengths and limitations of the review

This review undertook a comprehensive search of four databases, with assessment of bias and certainty of evidence assessed using Cochrane guidance [25]. All participants in included studies had at least one CVD risk factor as defined by NICE criteria. Studies were mostly conducted in primary healthcare settings, which can be applicable to the general populations [46]. Subgroup analysis identified the most effective DHI in reducing CVD risk as automated email messaging. As the result was inconclusive, larger studies may provide more definite answers. Only English-language studies were considered, meaning relevant trials could potentially have been excluded. Furthermore, only six studies were included – publication bias (widespread in modern meta-analyses [47]) could not be calculated.

The review focused on secondary prevention, particularly reducing CVD risk for patients in the primary care setting. In future as DHIs potentially integrate more significantly into clinical practice, digital interventions will likely first be offered to the highest-risk patients, i.e. patients with a previous major CVD event. This higher-risk patient population would potentially engage with and benefit from DHI interventions as part of secondary prevention.

Stratifying risk via clinically-validated, cardiovascular risk scores such as QRISK2 and the D’Agostino scale offers an efficient approach to evaluate trial results. Synthesised risk scores, which include factors such as ethnicity for QRISK2 [48] (now superseded by QRISK3 [20]), are useful tools in clinical practice to compile a patient’s individual risk scores, and are widely used in primary and secondary care.

Focusing on the individual components of CVD, such as systolic blood pressure or smoking and for primary prevention would offer another opportunity to evaluate targeted reduction strategies of CVD risk using DHIs. However, aggregated risk scores potentially cause over-simplification of their care, particularly for patients in the highest risk bracket [49]. Another specific limitation was use of Framingham 10-year risk score (FRS) as an outcome measure. Despite being reported in 4 included studies, FRS was developed in American white, middle-aged males—not validated for other populations—and studies in Primary Care shows FRS over-estimates CVD risk in British men [50], and underestimates risk in ethnic minorities [51]. Additionally, Salisbury’s study used QRISK2 as an outcome score [27], which has been replaced by QRISK3 – which adjusts for ethnicity, and other co-morbidities, on CVD risk [20]. There were concerns with bias regarding the measurement of outcome data as evaluated using the RoB-2 tool. For example, smoking history measurement was often inconsistent or unreported. Gonzalez-Sanchez’s [26] study considered only those who had smoked in the past year-to-date a smoker (excluding those who may have smoked over a year prior to the study – for any length of time and then quit). Inexact measurement of outcome results in information bias [52] which could reduce review validity. Measures to address this include quantitative bias assessment, through selecting variables in the data which may be subject to bias [53]. This could be achieved by selecting which assessing the potential quality of recorded results, considering sources of bias and assigning exact parameters to data where bias may arise.

Further secondary outcomes could be considered, for example to determine if DHIs can reduce hospitalisation rates for high-risk CVD patients. Further assessment on patient outcome by ethnicity, gender or age could yield useful results when considering appropriate treatment plans across sociodemographic boundaries. DHIs are likely to have lower uptake in older patients, as studies suggest technological literacy decreases with increasing age [54].

### Challenges before DHI implementation

Novel DHIs are currently not subject to quality assurance or safeguarding protocols. The MARS rating system was developed in response to this issue, offering quality scoring to new smartphone applications [55]. However, this standard is unlikely to be consistently applied globally – therefore patients face challenges finding reliable, effective DHIs.

Finally, delivering DHIs requires prospective patients to have internet access, usually via a smartphone or laptop computer. Marmot identified this as a financial barrier to lower-income populations who suffer digital exclusion [56]. Consequently, on both a national and global scale, digital poverty may propagate health and outcome inequalities.

### Implications for future

From a clinical perspective, high imprecision in mean difference and substantial heterogeneity between studies means these results cannot be considered strong evidence in favour of implementation. However, DHIs may have a place within the healthcare system. The pooled subgroup analysis of studies involving automated email interventions yielded results moderately in favour of DHIs to reduce CVD risk over usual care, in line with other studies [14]. Opportunistically, the nature of DHIs as a remote, automated software may reduce time burdens on clinicians [57], and conserve resources [58]. The results of the review, although suggestive of modest benefits particularly from SMS messaging, must be interpreted in the context of the quality of the trials, including potential risk of bias. Definite conclusions regarding DHIs and their role in secondary CVD prevention cannot yet be drawn.

Following global lockdowns caused by COVID-19, there is increased demand for remote telemedicine approaches to preventative treatment [59], which will likely persist in the post-pandemic era. Those with high CVD risk were encouraged to stay at home, or ‘shield’, to avoid infection risk [60] resulting in less engagement with clinical services [7], which is known to worsen health outcomes [61] and likely exacerbate health inequalities. In the long-term, DHIs may feature as components of preventative health approaches championed by national and international organisations. Key priorities include equity of access. The NHS Long-term Plan outlines funding towards ‘Digitally-enabled care’ initiatives [5], involving preventative health technologies. Furthermore, the WHO deployed a technological package – ‘HEARTS’ – in 14 pilot countries, targeting modifiable CVD risk factors [62]. These tools are simple to integrate and may lead to improved, standardised care, although the efficacy is still under evaluation. Promisingly, HEARTS may offer equity of access in lower- or middle-income countries. Further high-quality research is required to draw more solid conclusions regarding the efficacy of DHIs in reducing CVD risk.

### Conclusion

Findings suggest specific DHIs such as automated email messages may improve CVD risk outcomes, but were inconclusive for DHIs overall. Further research into specific DHI modalities is required, including newer technologies, particularly wearable DHIs which record CVD-related data such as blood pressure and heart rate with longer follow up [63]. It is crucial that health service providers consider effective DHI approaches which are acceptable, secure and enable equity of access to promote CVD prevention and optimise management for individuals in the future.

## Supplementary Information

Below is the link to the electronic supplementary material.

Supplementary file1 (DOCX 13 KB)

## Data Availability

All included studies are publicly-available and can be found from the references of this research paper.
