# Supplementary material for: Digital health RCT interventions for cardiovascular disease risk reduction: a systematic review and meta-analysis
Source: Health Technol (Berl). 2022 Mar 25;12(4):687–700. doi: 10.1007/s12553-022-00651-0 (PMC8947848; doi:10.1007/s12553-022-00651-0)
Supplement: Supplementary file 1 — Supplementary file1 (DOCX 13 KB) [file 12553_2022_651_MOESM1_ESM.docx]

**Supplementary information alongside submission**

**Title: Digital health RCT interventions for cardiovascular disease risk reduction: a systematic review and meta-analysis**

*PubMed & MEDLINE – search strategy*

(Cardiovascular disease* OR stroke OR myocardial infarction OR heart disease OR heart failure OR OR hyperten* OR arteriosclerosis OR atherosclerosis) AND (digital health OR digital technology OR telemedicine OR mobile health OR mhealth OR reminder systems OR ehealth OR health technology information OR text messaging OR telemedicine OR smartphone OR cell phones OR mobile applications OR reminder) AND (Smoking* OR Tobacco OR Blood pressure OR cholesterol OR Lipids OR cardiovascular risk OR Framingham OR QRISK* OR globorisk OR prevention)

*Cochrane Database of Systematic Reviews*

Search: cardiovascular disease digital health

Filters: Topic (heart and circulation), Date (01/01/2010 onwards)
